# 

**Published:** 1897-08

**Authors:** 


W. HORACE HOSKINS, D.V.S.,

H. D. GILL, V.S.

### CONTENTS.

ORIGINAL ARTICLES: PAGE

Some Aspects of Association Aims and Work. By W. HORACE HOSKINS, D.V.S. . . . 475
Colic. By E. P. SCHAFFTER, V.S. . . . . . . . . 482
Methyl-violet, or Pyoktanin. By W. HORACE HOSKINS, D.V.S. . . . . 484
The Floating Cartilage in Joints. By W. J. MARTIN, V.S. . . . . . 489

ABSTRACTS FROM FOREIGN JOURNALS:

Inoculations Against Swine-erysipelas (Rothlauf) . . . . . . . . 491
Sexual Communication Between a Dog and a Hen . . . . . . . 492
A New Stud Farm in Hungary . . . . . . . . . . 493
Diagnosis and Course of Moon-blindness in Army Horses . . . . . 493

REPORTS OF CASES:

I. Infectious Pneumonia. II. Tetanus. III. Diabetic Symptoms from Contaminated Drinking-water. By J. W. PETTY, D.V.S. . . . . . . . 495
Tetanus: The Use of Antitoxin. By C. Z. LABERGE, V.S. . . . . . . 496

U. S. V. M. A.—On to Nashville . . . . . . . . . . . 498

EDITORIALS:

Winnipeg a Good Example . . . . . . . . . . . 502
The Keystone State Takes an Important Step . . . . . . . 502
U. S. V. M. A. Proceedings . . . . . . . . . . . 503
The Antivivisection Bill in the U. S. Senate . . . . . . . 504
The Good Still Increases . . . . . . . . . . . 504
An Excellent Selection . . . . . . . . . . . 505
Why is it Thus . . . . . . . . . . . 506
Necrology . . . . . . . . . . . . . 506

REVIEW . . . . . . . . . . . . . . . 506

LEGISLATION:

New Jersey—Pennsylvania—Delaware—District of Columbia—Kentucky—Connecticut—Michigan—Kansas . . . . . . . . . . 511–515

CONTROL WORK:

Pennsylvania—Kansas—Ontario—National—Montana—New Hampshire—Mississippi . . . . . . . . . . . . . . 515–518

CORRESPONDENCE . . . . . . . . . . . . . 519
AMONG THE COLLEGES . . . . . . . . . . . . 523
BOARDS OF EXAMINERS . . . . . . . . . . . . 527
COMMENCEMENT EXERCISES . . . . . . . . . . . 528

SOCIETY PROCEEDINGS:

Synopsis of Meeting of Virginia State Veterinary Medical Association—Missouri Valley Veterinary Medical Association—Minnesota State Veterinary Medical Association—The North Carolina Veterinary Medical Association—The Pennsylvania State Veterinary Medical Association—Keystone Veterinary Medical Association . . . . . . . . . . . . . 528–536

AMONG THOSE YOU KNOW . . . . . . . . . . . 536
THINGS AND CONDITIONS ARE EVER CHANGING . . . . . . 539
GLEANINGS . . . . . . . . . . . . . . 540

